# Myostatin Knockout Mice Have Larger Muscle Fibers With Normal Function and Morphology

**DOI:** 10.1002/mus.28389

**Published:** 2025-03-03

**Authors:** Hans Degens, Ketan Patel, A. Matsakas

**Affiliations:** ^1^ Department of Life Sciences Manchester Metropolitan University Manchester UK; ^2^ Lithuanian Sports University Institute of Sport Science and Innovations Kaunas Lithuania; ^3^ School Biological Sciences University of Reading Reading UK

**Keywords:** capillary, force, microcirculation, myostatin, oxidative capacity, oxygenation, power, single fiber

## Abstract

**Introduction:**

We assessed whether muscle fibers in myostatin knockout (MSTN−/−) mice are just larger or also exhibit morphological, metabolic, and functional differences from MSTN+/+ mice.

**Methods:**

We compared single fiber contractile properties and histological fiber properties in muscles from MSTN−/− and MSTN+/+ mice.

**Results:**

Even though in permeabilized muscle fibers from the extensor digitorum longus and soleus muscle maximal force was higher (*p* < 0.001) there were no significant differences in specific power (power per unit volume), specific tension (force per cross‐sectional area), maximal shortening velocity, or curvature of the force‐velocity relationship between MSTN−/− and MSTN+/+ mice. In histological sections of the soleus muscle, fibers were larger (*p* < 0.001), but the succinate dehydrogenase staining intensity and capillary density did not differ significantly between MSTN−/− and MSTN+/+ mice, which was explicable by the larger number of capillaries around a fiber (*p* < 0.001). A model showed no significant differences in soleus muscle oxygenation.

**Discussion:**

The larger force‐generating capacity of fibers from MSTN−/− mice is explicable by the larger fiber cross‐sectional area. The data indicate that muscle fibers from MSTN−/− mice are quantitatively, but not qualitatively different from muscle fibers from MSTN+/+ mice. Myostatin inhibition may help increase muscle mass in conditions accompanied by muscle weakness without a detrimental impact on muscle quality, but systemic side effects need to be considered.

**Abbreviations**
CAF: capillaries around a fiber
CFD: capillary fiber density
EDL: *m. extensor digitorum longus*
FCSA: fiber cross‐sectional area
INT: integrated
LCFR: local capillary to fiber ratio
Log_R_SD: logarithmic standard deviation of domain radii
M: fraction of maximal shortening velocity or force at peak power
MHC: myosin heavy chain
MSTN: myostatin
OD: optical density
SDH: succinate dehydrogenase
*V*_max_
: maximal shortening velocity

## 1 Introduction

Myostatin puts a brake on developmental muscle growth as reflected by the larger muscle mass of mice with inactive, or knocked out, myostatin [1]. This has led to the exploration of the use of myostatin inhibitors in the treatment of Duchenne muscular dystrophy and other conditions associated with muscle weakness [2]. The inverse relationship between the cross‐sectional area and the maximal oxygen consumption of a muscle fiber is suggested to be determined by oxygen diffusion limitations and is dubbed the ‘size principle of striated muscle cells’ [3]. While part of the larger muscle mass is attributable to a larger number of muscle fibers, the muscle fibers of these animals are also larger than in normal mice [1, 4, 5]. At first glance, the hypertrophied fibers seem to defy the size principle, but this has been reported to be accompanied by a reduction in the number of oxidative fibers [6, 7] and hence the size principle may still be obeyed.

Although it has been argued that compensatory hypertrophy is made possible by angiogenesis [8, 9, 10, 11], the capillary to fiber ratio (C:F) may be lower, rather than higher, in myostatin knockout mice [5, 12]. Therefore, it is likely that the size principle is not obeyed by muscle fibers in muscles from mice with dysfunctional myostatin and that impaired muscle oxygenation may underlie the earlier exhaustion during treadmill running [7], and lower in vivo plantaris [13] and gastrocnemius [14] muscle fatigue resistance. However, the relationships between the size, oxidative capacity, and capillary supply in individual muscle fibers have hitherto not been evaluated, nor the potential impact on muscle oxygenation.

Even though muscles from myostatin knockout mice have been reported to have a larger force‐generating capacity, the specific tension (force per cross‐sectional area or mass) is lower [7, 14, 15]. This deficit may exist at the level of single fibers, suggesting that muscle fiber function is compromised [16]. However, little attention has been given to the shape of the force–velocity relationship, where not only maximal shortening velocity and force, but also the curvature (a/Po) of the force–velocity relationship are important determinants of power [17]. Finally, there may be a muscle‐specific effect, where, for instance, the specific tension was lower in the extensor digitorum longus (EDL), but not in the soleus muscle of myostatin knockout mice [7, 15].

The objective of the present study was therefore to assess whether myostatin knockout induces changes in muscle quality. We hypothesized, given the (a) apparent breaking of the size principle and (b) reported reductions in specific tension, that myostatin knockout not only results in (i) larger muscle fibers but also (ii) a lower specific power of individual muscle fibers that is attributable to both (a) a higher curvature (lower a/Po) of the force‐velocity relationship and (b) a lower specific tension. In addition, it is hypothesized that myostatin knockout will induce (iii) a lower oxidative capacity of, and capillary supply to, individual fibers that, combined with the larger fiber size, will result in (iv) impaired muscle oxygenation that may underlie the increased reliance on glycolytic ATP production during the later stages of a muscle fatigue test [14].

## 2 Methods

### 2.1 Animals

Male myostatin−/− (MSTN−/−) and wild type (MSTN+/+) mice (C57BL/6) were 6, 12, 18, or 24 months old at the time of sacrifice. The MSTN−/− mice were a gift from Se‐Jin Lee (John's Hopkins, USA) [1]. The mice had free access to food and water and were kept on a 12:12 h dark: light cycle. Animals were maintained and experiments performed under a license from the UK Home Office in accordance with the Animals (Scientific Procedures) Act 1986. The study was approved by the local ethics committee of the University of Reading. Animals were euthanized by cervical dislocation, and the soleus and EDL were excised and placed in ice‐cooled relaxing solution for determination of single fiber contractile properties, or frozen in isopentane cooled in liquid nitrogen for histochemistry.

### 2.2 Single Fiber Contractile Properties

The solutions have been described previously [17, 18, 19]. Relaxing solution (mmol L^−1^): MgATP, 4.5; free Mg^2+^, 1; imidazole, 10; EGTA, 2; KCl, 100; with the pH set to 7.0 using KOH. The low‐EGTA solution was the same as the relaxing solution except that EGTA was 0.5 mM. The pCa (−log[free Ca^2+^]) of the activating solution was 4.5 and contained MgATP, 5.3; free Mg^2+^, 1; imidazole, 20; EGTA, 7; creatine phosphate, 19.6; KCl, 64; pH 7.0.

The procedures for determining single fiber contractile properties were as described previously [17, 18, 19]. Briefly, muscle bundles were cut from each muscle and immersed in glycerol/relax at 4°C for 24 h. They were then sucrose‐treated, frozen in liquid nitrogen, and stored at −80°C for later use [20].

Fiber bundles were placed in a relaxing solution containing 1% Triton X‐100 for 20 min to permeabilise the membranes. Individual fibers dissected from the bundles were mounted in a permeabilized‐fiber test system (400, Aurora Scientific Inc., Aurora, Ontario, Canada) and tied with nylon thread to fine insect pins attached to the force transducer (Aurora, 403A) and motor arm (Aurora, 312C). These were mounted over a moveable stainless‐steel plate containing a set of machined wells, each with a glass base. The plate was cooled to 15°C, and the plate, transducer, and motor were mounted on an inverted microscope (Olympus IX71, Tokyo, Japan). The image of the fiber was viewed by a video camera, and sarcomere length was determined via a Fourier transformation of the sarcomere pattern (900A, Aurora). Sarcomere length was set at 2.6 μm at the start of the experiment and checked at regular intervals thereafter. Fiber diameter was measured at three places while suspended in the air, and fiber cross‐sectional areas (FCSA) calculated assuming the fiber attained a circular circumference [21]. Fiber length was measured to the nearest 0.01 mm.

Fibers were transferred from the relax solution to a low‐EGTA solution for 15 s before being moved to the well containing the activating solution (pCa 4.5). When the isometric force had reached a plateau, the fiber was subjected to four sequences of four isotonic shortening steps [17, 18, 22]. At the end of a sequence, the fiber was stretched back to its original length while still in the activating solution. This sequence was repeated four times at different percentages of isometric force. Each step in a sequence lasted 150 ms.

### 2.3 Data Analysis

The specific tension was given as maximal force per fiber cross‐sectional area, where the cross‐sectional area was corrected for 20% swelling. The data for force and length were analyzed by fitting least squares linear regressions to the last 100 ms of each step, generating a value for velocity of shortening for a given force and yielding 16 data points for each fiber. These data were fitted to the Hill equation: (*P* + *a*) (*V* + *b*) = (*P*_0_ + *a*)*b* [23] using a non‐linear least squares regression and an iterative routine written in Matlab (v7.1, The Mathworks Inc., Natick, Massachusetts, USA) to give best fit values for the Hill constants a and b, with *P* expressed as a fraction of *P*_0_. These values were used to estimate the maximum velocity of unloaded shortening (*V*_max_) as fiber lengths per second (FL s^−1^).

The fraction (*M*) of *P*_0_ or *V*_max_ at which peak power was generated was calculated as: *M* = (√(1 + *G*) − 1)/*G*, derived from the Hill equation, where *G* is *P*_0_/*a* or *V*_max_/*b* [24]. Maximum power was calculated as: *M* ^2^ × *P*_0_ × *V*_max_ and is reported as Watts per liter (W L^−1^).

Force data were accepted if a plateau was reached. Force‐velocity data were rejected if the isometric force decreased by more than 10% over the course of the four sequences of isotonic shortening contractions, the sarcomere length had changed by more than 0.1 μm or if the *R* ^2^ was less than 0.95 when fitting the data to the Hill equation. In a previous study, we determined that the coefficients of variation for *V*_max_, *a*/*P*_o_, and *P*_0_ are 9%, 7%, and 6%, respectively [17].

### 2.4 Gel Electrophoresis

The myosin heavy chain composition of the fibers was determined with polyacrylamide gel electrophoresis as described previously [17, 18, 19]. The stacking (4%) and separating (6%) gels contained 30% glycerol. Approximately 0.1 mm fiber length was loaded, and the gel was run for 27 h at 15°C. The gels were stained with a silver stain plus kit (Biorad, Hemel‐Hempstead, UK) and fibers were classified according to their migration distance on the gel. We were unable to separate the type II myosin isoforms, a problem also reported by others [25, 26] and have therefore only compared type I with type II fibers.

### 2.5 Histology

**FIGURE 1:**
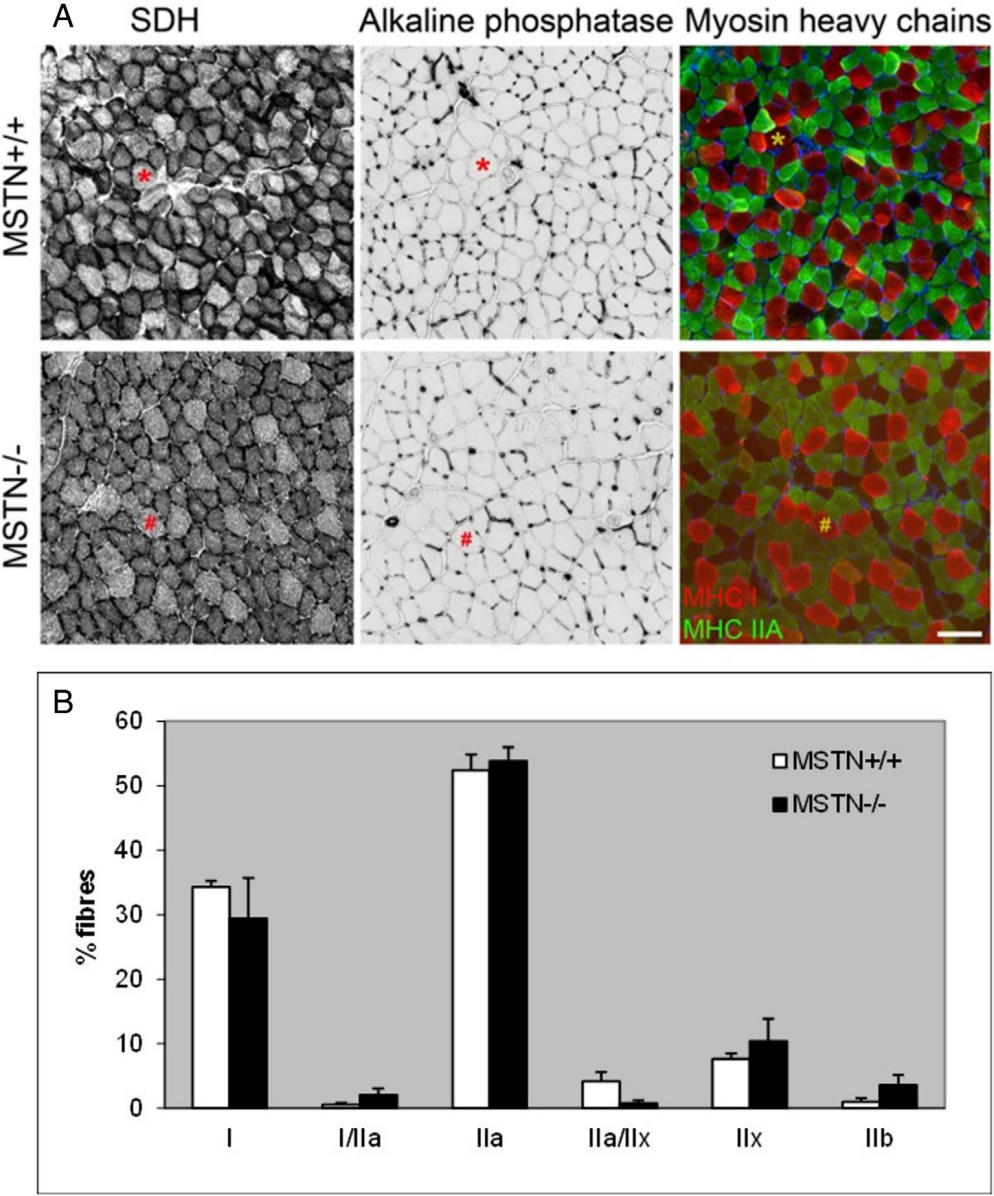
(A) Serial sections of the soleus from myostatin knockout (MSTN−/−) and wildtype (MSTN+/+) mice stained for succinate dehydrogenase (SDH), capillaries with alkaline phosphatase, and fiber type (myosin heavy chains). *Identical fiber in each picture. Scale bar 50 μm. (B) Fiber type composition in the soleus from MSTN+/+ and MSTN−/− mice. Values are mean ± SEM.

Serial 10‐μm sections of the soleus were cut on a cryostat and stained for succinate dehydrogenase (SDH), fiber type composition, and capillaries. An example of serial sections stained for SDH, capillaries, and fiber types is shown in Figure 1A. For each muscle, 74–114 fibers were analyzed.

To identify different fiber types, sections were stained with myosin heavy chain (MHC) type‐specific antibodies as described previously [4, 7]. Briefly, sections were permeabilized for 15 min in 20 mM HEPES, 300 mM sucrose, 50 mM NaCl, 3 mM MgCl_2_, 0.5% Triton X‐100, pH 7 at 4°C. This was followed by blocking in 5% fetal calf serum (v/v), 0.05% Triton X‐100 in phosphate‐buffered saline for 30 min. One section was stained for IIA and IIB MHC with A.474 mouse IgG (1:4) and BFF3 mouse IgM (1:1) monoclonal antibodies (Developmental Studies Hybridoma Bank, University of Iowa, Iowa city, IA, USA), respectively. A serial section was stained for type I MHC by A8.480 mouse immunoglobulin M (IgM, 1:1) from the Hybridoma bank. After overnight incubation at 4°C, type I and IIB MHC isoforms were detected with Alexa Fluor 633 goat‐anti‐mouse IgM (Molecular Probes A21046, 1:200) and type IIA MHC with Alexa Fluor 488 goat‐anti‐mouse IgG (Molecular Probes A11029, 1:200), respectively. Slides were mounted in Mowiol 4–88 (Calbiochem).

A serial section was stained for SDH by incubation for 3 min in 75 mM sodium succinate, 1.1 mM nitroblue tetrazolium, and 1.03 mM phenazine methosulphate in sodium phosphate buffer [7]. Another serial section was stained with alkaline phosphatase to identify capillaries [9].

### 2.6 Morphometry

The fiber cross‐sectional area (FCSA), fiber type composition, and measures of capillarisation were determined with the method of capillary domains [9]. A capillary domain is defined as the area surrounding a capillary, delineated by equidistant boundaries from adjacent capillaries [27] and is a good estimate of the capillary oxygen supply area [28]. The capillary domain method not only gives overall indices of muscle capillarisation, such as the capillary density (CD) and capillary to fiber ratio (C:F), but also the capillary supply to individual fibers.

Capillary and fiber outline coordinates are recorded, and the overlaps of fibers and capillary domains are assessed. For a given fiber, the sum of the domain fractions overlapping a fiber is presented as the local capillary‐to‐fiber ratio (LCFR) and the LCFR divided by FCSA gives the capillary fiber density (CFD). In addition, the heterogeneity of the distribution of capillaries is given as the standard deviation of the logarithm of the domain radius (Log_R_SD). The higher the Log_R_SD, the more heterogeneous the distribution of the capillaries, where a high heterogeneity has a negative impact on muscle tissue oxygenation [29, 30, 31, 32].

The capillary data were fed into a computer model (OxyTis, https://hoofd.info/louis/s/s_Apps.html) to determine the impact of myostatin knockout on tissue oxygenation of a muscle working at maximal oxygen consumption [33]. As before, the extraction pressure (steep gradient near the capillary) was 5 mmHg, myoglobin‐facilitated pressure 19 mmHg, and the Flow/Diffusion coefficient was set at 75 μm [32].

The SDH activity of each cell was determined with densitometry that for each section was corrected for background staining [7] and given as SDH OD. The product of FCSA × SDH OD was given as the Integrated SDH for a muscle fiber (SDH INT).

### 2.7 Statistics

Data were analyzed with SPSS version 28 (IBM, Armonk, NY, USA). An analysis of variance was used with a linear mixed‐effects model with group, muscle, and fiber type as fixed factors. Effects and interactions were considered significant at *p* < 0.05.

## 3 Results

The body and muscle masses, as well as the muscle mass: body mass ratios, were higher in the MSTN−/− than the MSTN+/+ mice, but there was no significant difference for the soleus muscle mass: body mass ratio (Table 1).

**TABLE 1:** Body mass (BM), and soleus (Sol) and extensor digitorum longus (EDL) muscle mass and muscle mass to body mass ratio in wild type (MSTN+/+) and myostatin knockout (MSTN−/−) mice.

	BM (g)	EDL (mg)	Sol (mg)	EDL/BM (mg g^−1^)	Sol/BM (mg g^−1^)
MSTN+/+ (5)	26.4 ± 0.9	8.98 ± 0.40	8.04 ± 0.67	0.339 ± 0.006	0.302 ± 0.016
MSTN−/− (7)	36.8 ± 1.4^a^	20.61 ± 0.86^a^	13.16 ± 0.72^a^	0.562 ± 0.018^a^	0.360 ± 0.022
Myostatin	*p* < 0.001	*p* < 0.001	*p* < 0.001	*p* < 0.001	NS

*Note*: Values are mean ± SEM; Between brackets: *n*.

^a^ Different from MSTN+/+.

### 3.1 Single Fiber Contractile Properties

As we did not observe any significant effects of age, we have pooled all fibers for further analysis. Of the 174 classified soleus and EDL fibers, one was a hybrid type I/II fiber (not included in the fiber type analysis), 10 were type I fibers and they came only from the soleus (4 MSTN+/+ and 6 MSTN−/−) and the remaining 163 fibers were type II fibers. Type I fibers had a higher specific tension than type II fibers (*p* = 0.047), but *V*_max_ (*p* = 0.032), *a*/*P*_0_ (*p* = 0.01), specific power (*p* = 0.048) and velocity at peak power (*V*_opt_) (*p* < 0.001) were all higher in type II than type I fibers. As there were no significant group × fiber type interactions, for the subsequent analysis, all fibers were pooled, which also allowed us to include unclassified fibers in the analysis, making the total 126 MSTN+/+ and 157 MSTN−/− fibers.

Soleus fibers had a higher specific tension and lower *V*_max_ and *V*_opt_, a/Po, and specific power than those of the EDL. There were no significant differences in specific tension at peak power (*P*_opt_) between the soleus and EDL (Table 2).

**TABLE 2:** Contractile properties of single fibers in the soleus (Sol) and extensor digitorum longus (EDL) muscles of wild type (MSTN+/+) and myostatin knockout (MSTN−/−) mice.

		CSA (μm^2^)	*P*_0_ (μN)	ST (N cm^−2^)	*V*_max_ (FL s^−1^)	Power (W L^1^)	*a/Po*	*V*_*opt*_ (FL s^−1^)	*Popt* (N cm^−2^)
Sol	MSTN+/+	1719 ± 63 (63)	164 ± 9 (63)	14.6 ± 0.9 (63)	0.87 ± 0.04 (51)	13.0 ± 1.5 (51)	0.24 ± 0.02 (51)	0.25 ± 0.01 (51)	4.65 ± 0.35 (51)
	MSTN−/−	2854 ± 157 (98)^a^	251 ± 11 (98)^a^	14.0 ± 0.5 (98)	0.88 ± 0.00 (65)	12.0 ± 0.8 (65)	0.22 ± 0.01 (65)	0.26 ± 0.01 (65)	4.43 ± 0.19 (65)
EDL	MSTN+/+	2012 ± 111 (63)	170 ± 10 (63)	12.9 ± 0.6 (63)^b^	1.16 ± 0.06 (17)^b^	21.9 ± 2.5 (17)^b^	0.36 ± 0.04 (17)^b^	0.38 ± 0.02 (17)^b^	5.33 ± 0.43 (17)
	MSTN−/−	2566 ± 142 (59)^a^	205 ± 14 (59)^a^	12.3 ± 0.7 (59)^b^	1.11 ± 0.18 (5)^b^	18.6 ± 6.1 (5)^b^	0.33 ± 0.06 (5)^b^	0.37 ± 0.08 (5)^b^	4.13 ± 0.73 (5)
Myostatin		*p* < 0.001	*p* < 0.001	NS	NS	NS	NS	NS	NS
Muscle		NS	NS	*p* = 0.012	*p* = 0.001	*p* < 0.001	*p* = 0.002	*p* < 0.001	NS
Muscle × myostatin		*p* = 0.038	*p* = 0.026	NS	NS	NS	NS	NS	NS

Abbreviations: CSA, fiber cross‐sectional area; Po, maximal force; ST, specific tension; *V*_max_, maximal unloaded shortening velocity. Values are mean ± SEM (*n*).

^a^ Different from MSTN+/+.

^b^ Different from soleus muscle.

Fibers of MSTN−/− mice were larger than those of MSTN+/+ mice and more so in the soleus (66% larger) than in the EDL (28% larger) as reflected by the genotype × muscle interaction (Table 2). Also, *P*_0_ was higher in the MSTN−/− than in MSTN+/+ mice, and more so in the soleus (53%) than in the EDL (21%) muscle (genotype × muscle interaction). There were, however, no significant differences in specific tension, *P*_opt_, *V*_max_, *V*_opt_, or *a*/*P*_0_ between fibers from MSTN+/+ and MSTN−/− mice (Table 2). While 81% and 27% of the soleus and EDL fibers of MSTN+/+ mice usable for determination of specific tension were also suitable for the determination of force –velocity characteristics, this was only 66% and 8.5% of the fibers from MSTN−/− mice.

### 3.2 Muscle Morphology

There was no significant difference in fiber type composition of the soleus muscle between the MSTN−/− and MSTN+/+ mice (Figure 1B). There were also no significant differences in the C:F, CD, Log_R_SD, standard deviation in FCSA (SDFCSA) or coefficient of variation in FCSA (data not shown) between muscles from the MSTN−/− and MSTN+/+ mice (Table 3).

**TABLE 3:** Capillary to fiber ratio (C:F), capillary density (CD), heterogeneity of capillary spacing (Log_R_SD) and standard deviation of fiber size (FCSASD) in the soleus of wild type (MSTN+/+) and myostatin knockout (MSTN−/−) mice.

	C:F	CD (mm^−2^)	Log_R_SD	FCSASD (μm^−2^)
MSTN+/+ (4)	1.73 ± 0.11	636 ± 54	0.092 ± 0.006	556 ± 39
MSTN−/− (4)	1.86 ± 0.23	582 ± 46	0.098 ± 0.003	624 ± 38

*Note*: Values are means ± SEM. Between brackets: *n*.

**FIGURE 2:**
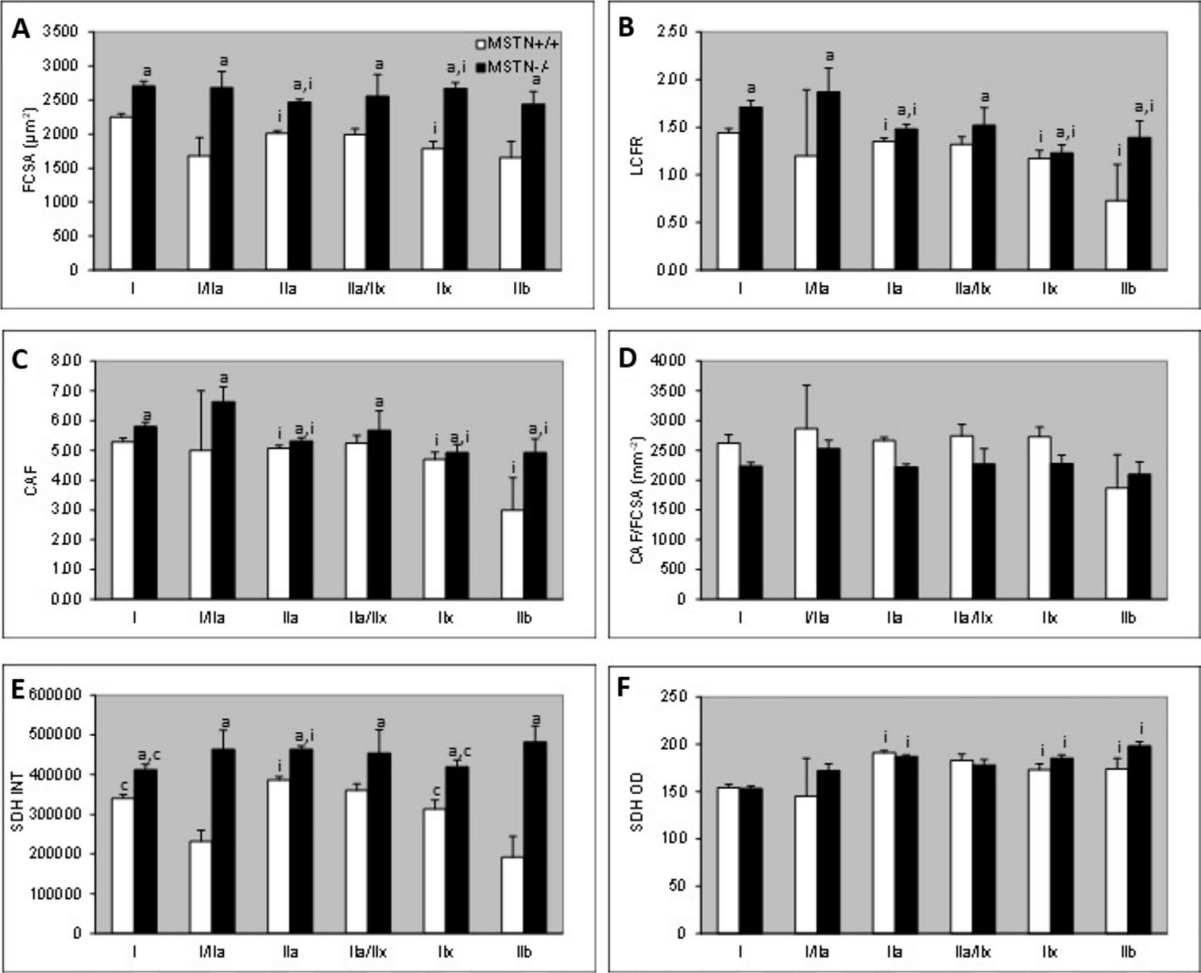
(A) Fiber cross‐sectional area (FCSA), (B) local capillary to fiber ratio (LCFR), (C) capillaries around a fiber (CAF), (D) CAF/FCSA, (E) optical density of succinate dehydrogenase (SDH OD) and (F) Integrated SDH (FCSA × SDH OD) of fibers of different types in the soleus from wildtype (MSTN+/+) and myostatin knockout (MSTN−/−) mice. Values are mean ± SEM. ^a^Main effect different from MSTN+/+ at *p* ≤ 0.005; ^i^: Main effect different from type I at *p* < 0.05; ^c^: Main effect different from type IIa at *p ≤* 0.007.

Irrespective of genotype, type I fibers were larger than type IIa and IIx fibers (Figure 2A), and had a higher LCFR (Figure 2B) and more capillaries around a fiber (CAF) (Figure 2C) than any other fiber type. There was no significant difference in CFD (data not shown) or CAF/FCSA between fiber types (Figure 2D). The SDH OD was lower in type I fibers than in any other fiber type (Figure 2E) and the SDH INT was higher in type IIa than type I and IIx fibers (Figure 2F).

There were no group × fiber type interactions, indicating that the effects of myostatin knockout were similar for all fiber types. The FCSA (Figure 2A), LCFR (Figure 2B) and CAF (Figure 2C) were higher for fibers from the MSTN−/− than those from the MSTN+/+ mice, but there was no significant difference between MSTN−/− and MSTN+/+ for CAF/FCSA (Figure 2D) and CFD (data not shown). Likewise, the higher SDH INT (Figure 2E) was explicable by the larger FCSA, but not associated with any significant difference in SDH OD between fibers from MSTN−/− and MSTN+/+ mice (Figure 2F).

**FIGURE 3:**
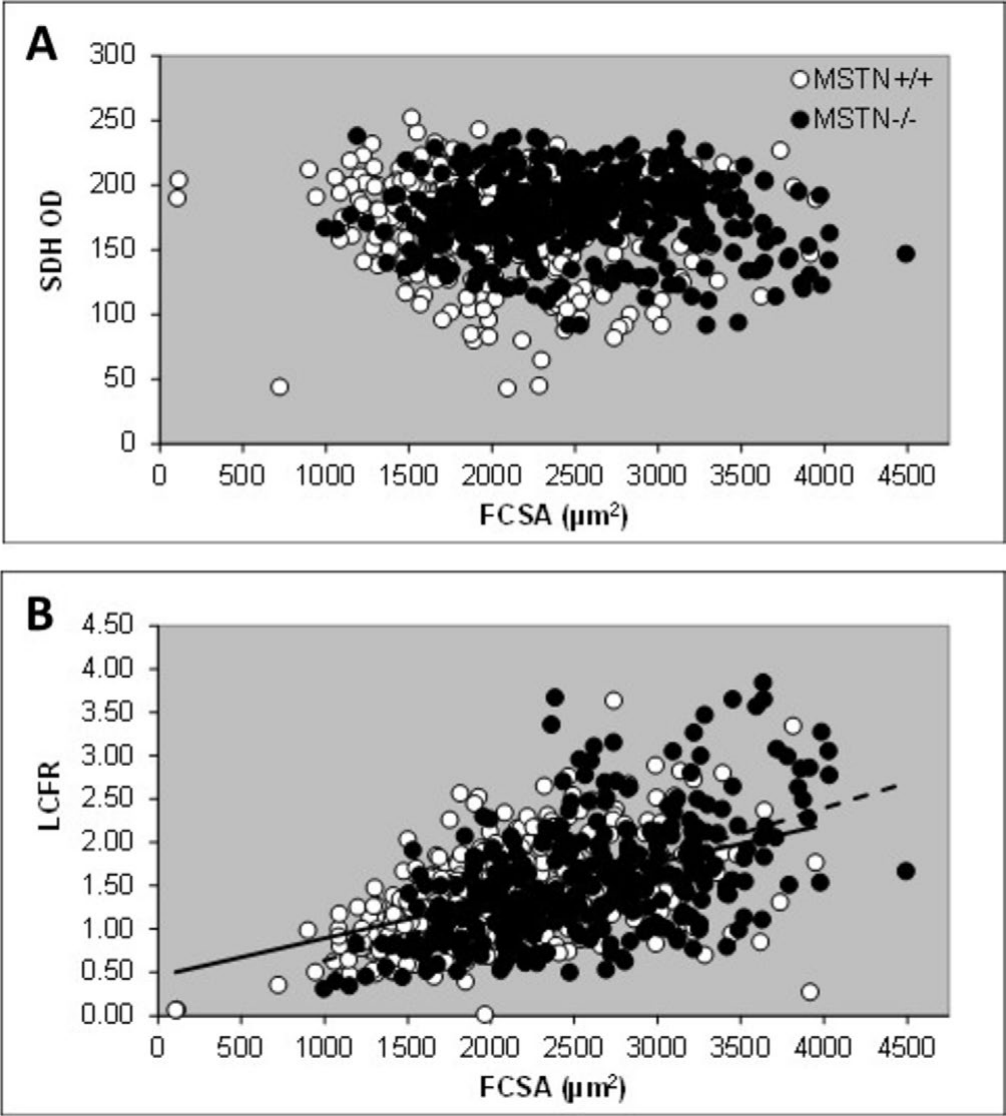
(A) optical density of succinate dehydrogenase (SDH OD) and (B) local capillary to fiber ratio (LCFR) vs. fiber cross‐sectional area (FCSA) in soleus muscle fibers from wildtype (MSTN+/+) and myostatin knockout (MSTN−/−) mice. Solid line: MSTN+/+; dashed line: MSTN−/−. *R* ^2^_adj_ = 0.281; *p* < 0.001.

There was no significant relationship between the SDH OD and FCSA of a fiber in either MSTN−/− and MSTN+/+ mice (Figure 3A). A stepwise regression revealed that the capillary supply to a fiber (LCFR) was primarily related to FCSA (*R* ^2^_adj_ = 0.281; *p* < 0.001) with a minor contribution of fiber type (*R* ^2^_adj_ = 0.288; *p* = 0.012) (Figure 3B for LCFR vs. FCSA). There was no significant contribution of SDH OD, nor genotype, indicating that the relationship was similar for muscle fibers from MSTN−/− and MSTN+/+ mice.

**FIGURE 4:**
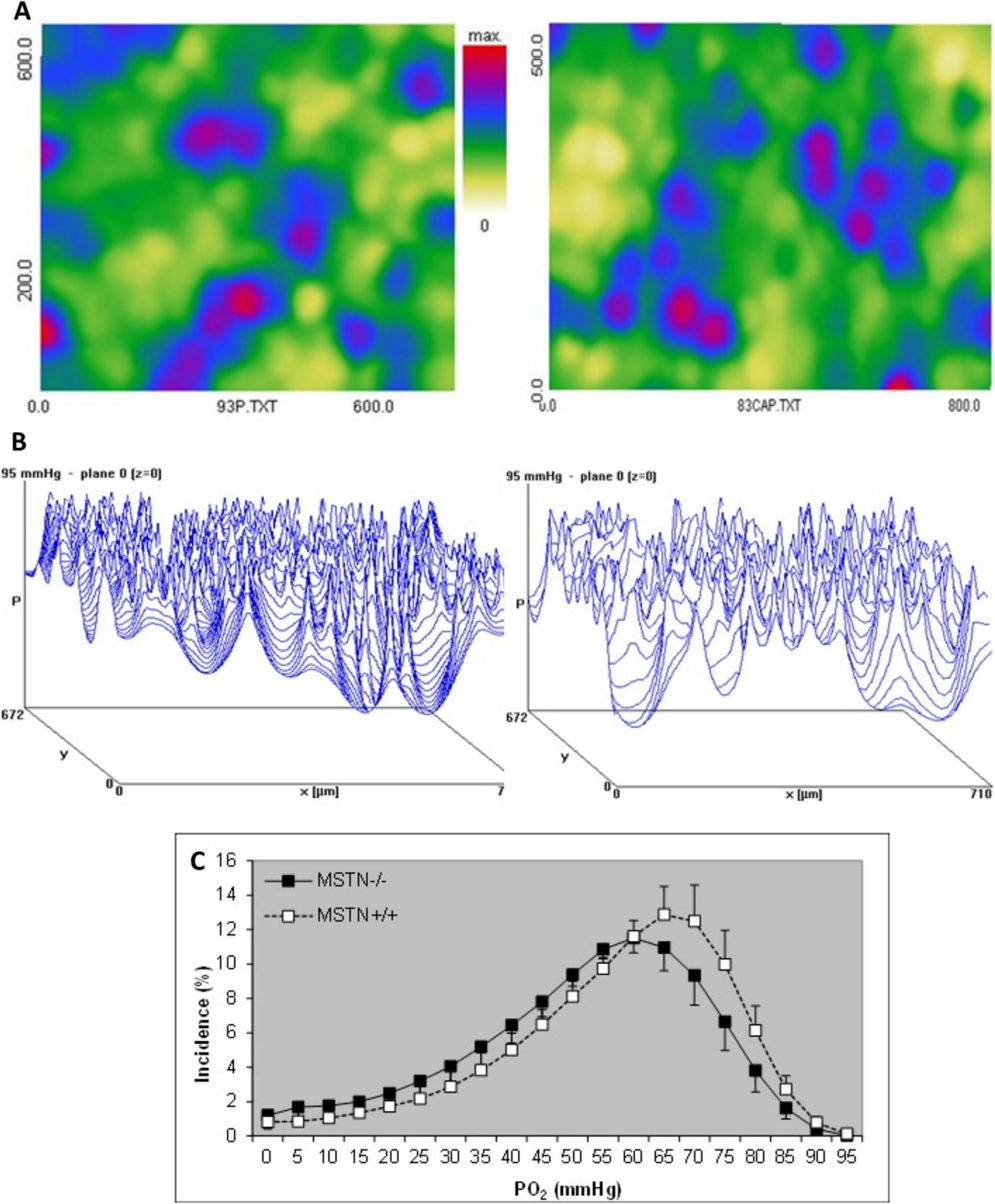
(A) Density distribution of capillaries and (B) tissue oxygenation in a MSTN+/+ (left) and MSTN−/− (right) mouse soleus muscle. The peaks of PO_2_ are the capillaries and the troughs are in the working muscle fibers. (C) PO_2_ distribution in soleus muscle from MSTN+/+ and MSTN−/− mice working at maximal oxygen consumption. Values are mean ± SEM.

Even though the MSTN−/− mice have larger fibers, muscle oxygenation (average PO_2_ MSTN−/− 49.5 ± 3.4 vs. MSTN+/+ 53.9 ± 4.5 mmHg) does not differ significantly from that seen in MSTN+/+ mice (Figure 4).

## 4 Discussion

The main observations are that the larger force‐generating capacity of soleus and EDL muscle fibers from myostatin knockout than those from wild‐type mice is due to their larger size, without significant differences in specific tension or the force–velocity relationship. Similarly, the larger number of capillaries per fiber and total oxidative capacity of a fiber were attributable to the larger fibers and not to a difference in mass‐specific oxidative capacity. There was also no significant difference in muscle oxygenation. Thus, even though myostatin knockout mice have larger fibers, they are metabolically and functionally qualitatively similar to fibers from normal mice.

### 4.1 Muscle Fiber Size and Fiber Type Composition

In line with reports that myostatin dysfunction results in an increased muscle mass and fiber size [1, 4, 6, 7, 13, 34, 35] we saw here that MSTN−/− mice had larger fibers compared to MSTN+/+ mice. Myostatin dysfunction also may induce a fast‐to‐slow shift in fiber type composition in the EDL but not in the soleus muscle [16, 35], as we observed.

### 4.2 Single Fiber Contractile Properties

Our observation that the specific tension of single fibers was similar in MSTN−/− and MSTN+/+ mice is at odds with the lower specific tension in the in vitro EDL [7, 15, 34] and in vivo plantaris [13] and gastrocnemius [14] muscles, but corresponds with the similar specific tension reported in the EDL of MSTN−/− and MSTN+/+ mice [36], and knockout or wild type rats [35]. As the specific tension of the in vitro soleus muscle is similar in MSTN−/− and MSTN+/+ [15, 36], it is possible that the lower specific tension of the pennate muscles from MSTN−/− mice is attributable to a larger pennation angle [37]. Nevertheless, others using single fibers have seen, in contrast to our observation, a lower specific tension of fibers from the EDL from MSTN−/− mice [16, 38], but not in fibers from the soleus [16] or the tibialis anterior muscle of MSTN−/− rats [35]. Although this suggests that the impact of MSTN−/− on specific tension is muscle‐ or fiber type‐specific, we found similar effects of myostatin dysfunction in type I and type II fibers. Overall, the data suggest that MSTN−/− has no significant impact on fiber specific tension.

Power is the product of force and velocity. Hence, the maximal shortening velocity (*V*_max_) and the maximal isometric force are important determinants of power. It is often ignored, however, that power is also determined by the curvature of the force–velocity relationship, where a large curvature (low *a*/*P*_o_)—all else being the same—results in a low power, and a small curvature (high *a*/*P*_o_) results in high power [17].

It has been reported that the specific power of single fibers from the EDL is less in MSTN−/− than those from MSTN+/+ mice [38]. The *V*_max_, like our observation, was similar, and the authors argued that the shape of the force –velocity relationship was also similar. However, a closer look at their Figure 3 shows that the MSTN−/− fibers would have a larger curvature if the velocity was expressed as a proportion of *V*_max_. The higher curvature and lower specific tension would contribute to the lower specific power of fibers from MSTN−/− mice. Even so, the effect was expected to be small, and perhaps not significant, in that study, which corresponds with the similar *a*/*P*_0_ in our study, and together with the similar *V*_max_ indicates that the cross‐bridge kinetics of muscle fibers are not significantly affected by MSTN−/−, as also suggested by others [16].

### 4.3 Muscle Fiber Oxidative Capacity and Capillarisation

We were surprised that the oxidative capacity (SDH OD) was higher in type II than type I fibers, but we were reassured by similar observations by others [16].

In contrast to many reports of a lower oxidative capacity in muscles from MSTN−/− mice [6, 7, 34, 39], we did not observe a lower staining intensity for SDH. The previous studies mainly concluded that there was a lower oxidative capacity based on the proportion of SDH+ and SDH− fibers [6, 7], but did not report the intensity of the staining in individual fibers. In the plantaris muscle, there was, like our study, no difference in the oxidative capacity of individual fibers from MSTN−/− and MSTN+/+ mice [13]. Yet, the total oxidative capacity (SDH INT) was higher for fibers from MSTN−/− than for those from MSTN+/+ fibers, indicating that the increase in fiber size was accompanied by a proportional increase in oxidative capacity. In line with this, it has been reported that the capacity for oxidative ATP synthesis was similar in the gastrocnemius of MSTN−/− and MSTN+/+ mice [14].

The capillary supply to a fiber is primarily determined by fiber size [40, 41] and during compensatory hypertrophy, the increase in fiber size is accompanied by angiogenesis [8, 13, 41, 42, 43]. Yet, it has been reported that MSTN−/− mice have a lower, rather than a higher number of capillaries per fiber (C:F) and capillary density, at least in the EDL [5, 12]. Similar to our observations in the soleus muscle, in the plantaris muscle, there was an elevated C:F that was—given the similar capillary density—in proportion to the larger fiber size [13], suggesting that the relationship between fiber size and capillary supply is maintained in MSTN−/− mice.

Even with a similar capillary fiber density, the larger diffusion distances from the capillaries on the periphery to the center of the fiber in MSTN−/− mice may have led to oxygen diffusion limitations [3], that may be further aggravated if the heterogeneity of capillary spacing is increased [29, 31]. Here we saw, however, that the heterogeneity of capillary spacing was similar in soleus muscles from MSTN−/− and MSTN+/+ mice. This indicates that the extra formation of capillaries was not only sufficient to prevent a lower capillary density but was also non‐random to maintain the heterogeneity of capillary spacing, ensuring adequate muscle oxygenation.

Particularly, EDL fibers from MSTN−/− mice appeared susceptible to damage, as only 8.5% of the fibers usable for determination of specific tension were also suitable to extract force‐velocity data, vs. 27% of the EDL fibers from MSTN+/+ mice (for the soleus this was 81% vs. 66%). Yet, the similar standard deviation of fiber size [1] also seen in our study suggests that there was no severe muscle degeneration.

### 4.4 Limitations

There were relatively few EDL fibers in which we could determine the force‐velocity relationship. Although our permeabilized single fiber preparation does not provide information on calcium handling and the integrity of the neuromuscular junction, the myostatin inhibitor follistatin has been shown to enhance, rather than diminish, neuromuscular integrity and in vivo force production [44]. It would have been interesting to extend our morphological observations to the EDL, as MSTN−/− has been shown to have muscle‐specific effects on hyperplasia and hypertrophy [4] that may extend to muscle‐specific responses in oxidative capacity and capillarization.

## 5 Conclusion

The larger force and power generating capacity of the soleus and EDL muscle fibers of MSTN−/− mice are attributable to the larger size of the fibers without a significant increase (or decrease) in specific tension and specific power. In addition, soleus muscle fibers from MSTN−/− and MSTN+/+ mice had a similar oxidative capacity, capillary density, and muscle oxygenation. In conclusion, myostatin dysfunction only increases the quantity, but does not negatively affect the quality of skeletal muscle fibers, holding promise for the application of myostatin inhibitors for the treatment of conditions associated with muscle wasting and weakness, such as Duchenne muscular dystrophy [2].

We confirm that we have read the Journal's position on issues involved in ethical publication and affirm that this report is consistent with those guidelines.

## Author Contributions

**Hans Degens:** conceptualization, investigation, writing – original draft, methodology, validation, visualization, writing – review and editing, software, formal analysis, project administration, data curation, supervision, resources. **Ketan Patel:** conceptualization, investigation, methodology, writing – review and editing, project administration, supervision, resources. **A. Matsakas:** conceptualization, investigation, methodology, validation, visualization, writing – review and editing, formal analysis, project administration, data curation, supervision, resources.

## Disclosure

The authors have nothing to report.

## Conflicts of Interest

The authors declare no conflicts of interest.

## Data Availability

The data that support the findings of this study are available from the corresponding author upon reasonable request.
